# Blood Trihalomethanes and Human Cancer: A Systematic Review and Meta-Analysis

**DOI:** 10.3390/toxics13010060

**Published:** 2025-01-16

**Authors:** Miaomiao Fu, Pengyu Xue, Zhuorong Du, Jingsi Chen, Xiaojun Liang, Jiafu Li

**Affiliations:** 1 School of Public Health, MOE Key Laboratory of Geriatric Diseases and Immunology Suzhou Medical College, Soochow University, Suzhou 215123, China; fumiaomiaofern@163.com (M.F.); jhxyzk@126.com (P.X.); zhuorongdu@163.com (Z.D.); jschen1993@suda.edu.cn (J.C.); 2 Kunshan Center for Disease Control and Prevention, Suzhou 215301, China

**Keywords:** trihalomethanes, blood, cancer, chloroform

## Abstract

The control of waterborne diseases through water disinfection is a significant advancement in public health. However, the disinfection process generates disinfection by-products (DBPs), including trihalomethanes (THMs), which are considered to influence the occurrence of cancer. This analysis aims to quantitatively evaluate the relationship between blood concentrations of THMs and cancer. Additionally, the relationship between blood chloroform concentration and cancer is analyzed separately. Following PRISMA guidelines, we conducted a thorough search in the PubMed, Web of Science, and CNKI databases. Statistical analysis was performed using Review Manager 5.4 software. After screening, seven studies meeting the evaluation criteria were included. A total of 1027 blood samples from patients with cancer and 7351 blood samples from the control group were collected. The average concentration of THMs in the blood of the experimental group was 46.71 pg/mL, while it was 36.406 pg/mL in the control group. The difference between the two groups was statistically significant (SMD = −0.36, 95% CI: −0.45 to −0.27, *p* < 0.00001). However, due to the limited research data on the relationship between blood THMs and cancer, the conclusions drawn exhibit high heterogeneity. Additionally, we discussed the carcinogenic mechanisms of THMs, which involve multiple biological pathways such as oxidative stress, DNA adduct formation, and endocrine disruption, with variations in accumulation and target sites potentially leading to different cancer types, for which evidence is currently lacking. In the future, further epidemiological and animal model studies on THMs should be conducted to obtain more accurate conclusions.

## 1. Introduction

In the 19th century, people gradually became aware of the potential for water pollution to cause and spread diseases, leading to an active search for methods to purify water [1,2]. The introduction of chlorination as a disinfectant in the early 20th century marked a significant milestone in public health, drastically reducing waterborne diseases such as cholera and typhoid [3]. However, in the 1970s, researchers such as Rook [4] and Bellar [5] first identified trihalomethanes (THMs) in chlorinated drinking water, marking the discovery of disinfection by-products (DBPs) and drawing widespread attention [6]. DBPs are formed during the water disinfection process when naturally occurring organic matter reacts with disinfectants such as chlorine, chloramine, and ozone through oxidation, addition, and substitution reactions [7]. Chlorinated DBPs are produced during pre-chlorination or post-chlorination disinfection processes when chlorine reacts with organic matter in the water. When chlorine is added to water, the reaction is as follows: Cl_2_ + H_2_O → HClO + HCl [8]. Free chlorine acts as both a moderately strong oxidizing agent and an electrophilic addition reagent, reacting with natural organic matter in water to produce compounds like THMs [9]. THMs are dominant DBPs formed from the reaction between organic/inorganic substances in the water and chlorine disinfectants [10]. They include trichloromethane (TCM), bromodichloromethane (BDCM), dibromochloromethane (DBCM), and tribromomethane (TBM) [7]. In 1976, the National Cancer Institute confirmed the carcinogenicity of chloroform through oral experiments on rats and mice [11]. The same year, the Environmental Protection Agency (EPA) published findings on the widespread presence of THMs in chlorinated drinking water [12]. The EPA classifies chloroform as a possible human carcinogen, based on animal studies demonstrating that exposure to cytotoxic doses may lead to liver and kidney tumors via short-lived toxic intermediates [13].

THMs are known to increase cancer risk through different exposure pathways, and the incidence of specific cancers varies based on the route of exposure and the type of tissue affected [14]. The gastrointestinal tract, respiratory system, and skin are the primary exposure pathways [10,15]. Studies have consistently linked higher THM exposure levels (typically above 60 μg/L) to an increased incidence of bladder cancer, particularly through the ingestion of chlorinated water [10]. This is thought to be due to the prolonged exposure of bladder tissue to concentrated metabolites of THMs excreted in urine [16,17]. Moreover, research indicates that dermal and inhalation exposures result in a significantly higher systemic absorption of THMs, particularly brominated THMs [14,18]. For example, individuals exposed to BDCM via dermal contact had maximum blood concentrations approximately 35 times higher than those who ingested the same amount orally, underscoring the significant impact of bypassing first-pass metabolism [19]. Similarly, epidemiological studies have found that the odds of bladder cancer were markedly higher for individuals with extended exposure to THMs through activities like showering or bathing, with an odds ratio of 1.83 compared to 1.35 for those with high ingestion levels [20]. These findings suggest that dermal and inhalation exposure routes may play a more prominent role in the systemic absorption and associated cancer risks of THMs. Considering these exposure pathways, the potential risk of THMs contributing to skin cancer and lung cancer is concerning, although current evidence remains insufficient to establish a definitive link [21].

Recently, many countries have incorporated THMs into their drinking water quality standards [22,23]. The United States, for example, set strict regulations for disinfection by-products in its 2001 National Primary Drinking Water Regulations [24]. The Maximum Contaminant Level (MCL) represents the highest permissible concentration of contaminants in public water systems. The MCL Goal (MCLG) is the maximum concentration at which no known or anticipated adverse effects on public health would occur [25]. MCLs are enforceable standards, whereas MCLGs are non-enforceable health goals. Although some contaminants do not have MCLGs, specific pollutants have designated MCLs: for example, bromodichloromethane (0), bromoform (0), and dibromochloromethane (0.06 mg/L) [26].

Blood THMs serve as biomarkers of internal exposure, offering a direct measure of the body’s burden of these compounds [27]. They provide a more accurate assessment of individual exposure compared to environmental measurements or self-reported water consumption data, thus potentially clarifying the relationship between THM exposure and cancer risk [28,29]. To date, much research has focused on confirming the carcinogenicity of THMs, with animal studies supporting their carcinogenic potential. However, further investigation is needed to understand the link between human exposure to THMs and cancer. Many studies have analyzed THMs in drinking water and assessed health risks, but these evaluations are often based on drinking water THMs rather than blood THMs [30,31,32]. This paper employs a meta-analysis to synthesize and analyze data from multiple studies to derive a comprehensive understanding of the relationship between blood THM concentrations and cancer. Furthermore, it discusses the carcinogenic mechanisms of THMs, aiming to provide insights for future research.

## 2. Methods

A systematic review was conducted following PRISMA guidelines, ensuring methodological rigor and comprehensive analysis [33]. The Prospero protocol, CRD42024628026, was followed.

### 2.1. Database Selection

The databases selected for this study are PubMed, Web of Science, and China National Knowledge Infrastructure (CNKI). The advantages of PubMed include the following:

1. High academic authority: PubMed, managed by the U.S. National Library of Medicine (NLM), is a free search engine for life sciences and medical research. It contains a vast collection of academic journal articles, research reports, and papers in the fields of life sciences and medicine, ensuring high academic quality and credibility.
2. Free access and timely updates: users can freely access the resources available on PubMed, making it easier to obtain the latest research findings and academic advancements in the medical and life sciences fields.
3. Wide coverage: PubMed collects research from around the world in the life sciences and medical fields, covering multiple disciplines, including basic medicine, clinical medicine, biomedical engineering, biochemistry, biotechnology, and more.
4. Powerful search and filtering features: users can search for and filter the literature using various criteria such as keywords, authors, journals, and publication dates, enabling them to quickly find the information they need.

CNKI is one of the largest comprehensive academic database platforms in China. It allows users to search for the relevant domestic literature in Chinese and provides academic evaluation tools that facilitate academic research and assessment. Web of Science, offered by Clarivate Analytics (formerly Thomson Reuters), is a comprehensive academic database platform that covers a wide range of disciplines, including the natural sciences, social sciences, and humanities. It offers a wide range of academic information to researchers from different fields.

Additionally, Web of Science provides various analytical tools such as citation analysis, author analysis, and journal analysis, helping users conduct in-depth analyses of academic research and trends.

By selecting research from these three databases, we can not only obtain comprehensive data but also gain insights into the current state of research both domestically and internationally, thus broadening perspectives for future research.

### 2.2. Literature Collection

A comprehensive literature search was conducted across three electronic databases—PubMed, CNKI, and Web of Science—to identify studies evaluating the association between trihalomethanes (THMs) and cancer risk. The search strategies incorporated a combination of controlled vocabulary (e.g., MeSH terms) and free-text terms related to trihalomethanes, cancer, and blood to ensure broad coverage of relevant studies.

### 2.3. Literature Screening and Evaluation

**Figure 1:**
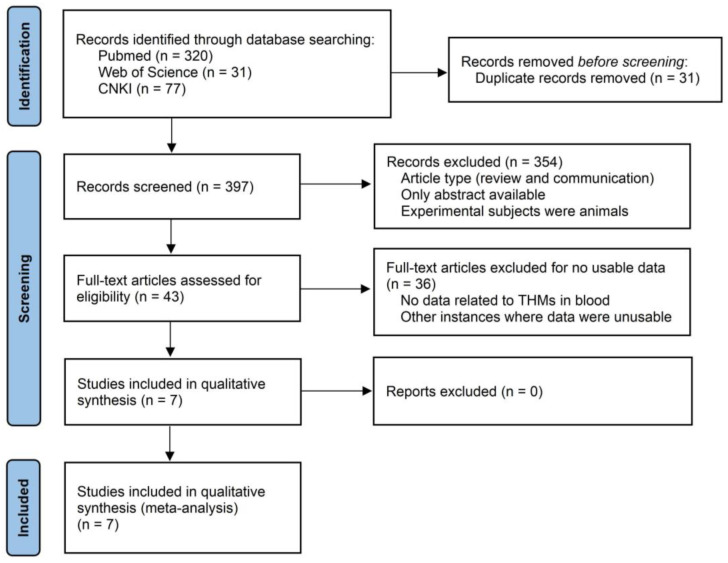
Flow chart of included studies (n = 7).

Two authors (M. Fu and P. Xue) separately gathered data from the chosen literature and performed cross-checks. In case of any discrepancies, they consulted with the third author (Z. Du) to reach a consensus. After the three databases were searched using the aforementioned search strategy, the following selection criteria were used: (1) time range—from database inception to April 2024; (2) content—As shown in Figure 1, a total of 428 related articles were initially obtained. However, the literature screening process did not stop there. First, 31 duplicate articles were removed based on their titles. Next, we conducted a preliminary review of the remaining articles to further refine the selection. We excluded articles that only provided abstracts, lacked sufficient data, did not focus on THMs in blood (such as studies on THMs in urine), involved animal subjects, or did not establish a connection between the results and cancer. In the end, 7 articles were retained for further analysis.

### 2.4. Visualization Analysis

This study utilized VOSviewer version 1.6.20, a software tool designed for constructing and visualizing scientific landscapes based on bibliometric networks. VOSviewer’s capabilities enable the development of evidence-based knowledge networks, highlighting research field developments, global collaborations, research hotspots, and emerging topics [34,35]. To understand the general research content of the literature, we analyzed titles and abstracts to identify the most frequently co-occurring terms. This analysis provided an overview of fundamental concepts and themes, thereby evaluating the knowledge framework of the field under study.

**Figure 2:**
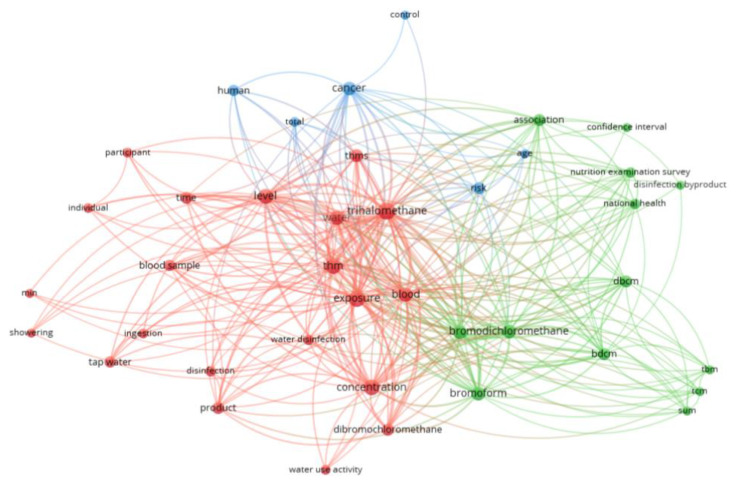
Co-occurrence network visualization map based on “title–abstract”.

Figure 2 presents a co-occurrence network visualization based on the “title–abstract” data. A minimum occurrence threshold of 5 was set for terms (binary counting: assessing the presence or absence of terms in document titles and abstracts, while ignoring the frequency of their occurrence). Out of 1077 terms, 40 terms met the established threshold (with a relevance score of 60%). The terms were categorized into three major clusters: Cluster 1 (red, 21 terms), Cluster 2 (green, 13 terms), and Cluster 3 (blue, 6 terms). The visualization was created using VOSviewer version 1.6.20. From Figure 2, it is evident that terms such as trihalomethane/THMs/THM, blood, and cancer are interconnected, showing a strong association and a high frequency of occurrence.

**Figure 3:**
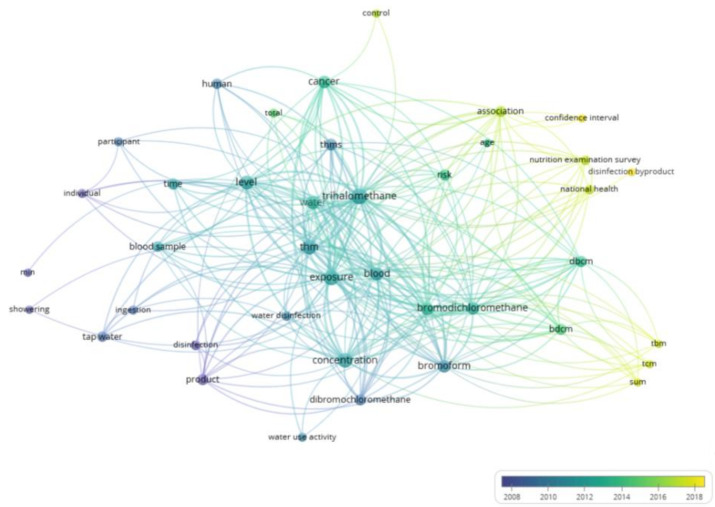
Co-occurrence visualization map of labels based on “title–abstract”.

Figure 3 shows a label co-occurrence visualization based on “title–abstract” data. A minimum occurrence threshold of 40 was set for terms (binary counting: evaluating the presence or absence of terms in document titles and abstracts, while ignoring their frequency within the documents). Out of 1077 terms, 40 terms met the established threshold (with a relevance score of 60%). Keywords were categorized by the publication year to better understand the characteristics of the research field. Blue nodes represent earlier research events, while yellow nodes represent more recent events. The visualization was created using VOSviewer version 1.6.20. From Figure 3, it is evident that research focus on trihalomethanes has shifted over time. Initially, the emphasis was on exposure factors such as drinking water and showering, but recent studies have increasingly concentrated on the health impacts of different concentrations of specific trihalomethanes in blood, particularly their carcinogenic effects.

### 2.5. Statistical Analysis

#### 2.5.1. Study Characteristics

As summarized in Table 1, among the 7 studies included [36,37,38,39,40,41,42], 2 were case–control studies, 3 were cross-sectional studies, and 2 were cohort studies. A total of 27,369 participants were involved, with sample sizes ranging from 933 to 12,173. These studies were published between 2008 and 2023, with 3 studies from the United States, 3 from Spain, and 1 from China.

#### 2.5.2. Study Subjects

This analysis involved 27,369 participants. In accordance with ethical principles, all participants provided informed consent. Most studies obtained participant lists and data from case investigations or databases, followed by interviews to assess age, gender, height, weight, and other factors. Researchers gathered information on participants’ daily behaviors, such as smoking and alcohol consumption, and assessed the presence of underlying conditions like hypertension and heart disease. During the interviews, samples were re-screened according to specific research criteria. For example, in the study “Bromodichloromethane Exposure and Non-Melanoma Skin Cancer”, participants were re-screened based on the following criteria: (1) age < 20 years, (2) lack of cancer prevalence data, (3) having cancers other than non-melanoma skin cancer, and (4) absence of THM blood concentration data [40]. This secondary screening helps avoid unnecessary work and reduces bias in subsequent research results, thus enhancing the accuracy and credibility of the findings. Some studies also divided participants into different cohorts based on certain conditions and conducted regular follow-up investigations. Despite differing study designs, all studies provided valuable conclusions.

#### 2.5.3. Risk of Bias Assessment

A bias risk assessment is crucial in meta-analysis and includes several aspects:

1. Study Quality Assessment: evaluates the quality level of the included studies.
2. Result Credibility: assesses the credibility of the meta-analysis results.
3. Conclusion Reliability: ensures the reliability and stability of conclusions.
4. Clinical Practice Guidance: provides valuable information for clinical decision-making.
5. Research Improvement: offers directions for future research improvements.
6. Scientific Research Standards: promotes the standardization and normalization of scientific research.

In this study, 2 case–control studies and 2 cohort studies were evaluated using the Newcastle–Ottawa Scale (NOS), while 3 cross-sectional studies were assessed using the Agency for Healthcare Research and Quality (AHRQ) criteria [43]. Two authors (Z. Du and M. Fu) evaluated the quality of the studies included in the research. In case of any discrepancies, they consulted with the third author (P. Xue) to reach a consensus. The AHRQ criteria for cross-sectional studies consist of 11 items, with each item scored as “1” for “Yes” and “0” for “No” or “Unclear”. The total score ranges from 0 to 3, 4 to 7, and 8 to 11, corresponding to low, medium, and high quality, respectively. An NOS score above 6 indicates high quality, while a score of 3 or below indicates low quality. Among the 2 case–control studies, one received a score of 7 (high quality), and the other received a score of 5 (medium quality). Of the 2 cohort studies, one received a score of 7 (high quality), and the other received a score of 6 (medium quality). Among the 3 cross-sectional studies, 2 were rated as medium quality (5–7 points) and 1 was rated as high quality (8 points) based on the AHRQ criteria.

#### 2.5.4. Blood Sample Collection

Whole blood samples were collected in gray-top glass vacuum containers containing potassium oxalate and sodium fluoride, unless otherwise specified (e.g., fasting requirements or specific sampling times). To prevent potential contamination, commercial vacuum containers were specially modified by laboratory personnel to remove most measurable volatile organic compounds (VOCs) [44]. Due to the high volatility of trihalomethanes (THMs), blood was drawn via venipuncture and stored at 4 °C during storage and transportation [45]. Detailed laboratory methods for analyzing blood THMs have been previously reported [44]. Briefly, the concentrations of TCM, BDCM, DBCM, and TBM in blood were determined using solid-phase microextraction gas chromatography–mass spectrometry [46], with data available in NHANES laboratory data files. The concentration of Br-THMs was calculated by summing the concentrations of BDCM, DBCM, and TBM, while blood THMs were calculated by summing TCM and Br-THMs [47]. Samples below the limit of detection (LOD) (range: 0.6–2.1 pg/mL) were assigned values of LOD/√2. To maintain the integrity of laboratory measurements, NHANES implemented a comprehensive data quality assurance program, which includes analyzing quality control samples at the beginning and end of each analytical run [36,44]. If the quality control results for a specific analyte were deemed “out of control”, data from all samples analyzed during that run were considered invalid.

#### 2.5.5. Covariates

Covariates were obtained through questionnaires during interviews and include variables related to the outcome of but not the primary focus of the study, such as age, gender, race/ethnicity, household income, education level, smoking, alcohol consumption, self-reported chronic diseases (e.g., diabetes, cancer, chronic obstructive pulmonary disease, hypertension, and cardiovascular disease), and general health status (excellent, very good, good, fair, or poor). To capture potential peak exposure events close to the time of blood sampling, participants also reported their water use activities in the preceding 72 h (e.g., swimming pools, hot tubs, or steam rooms) and the time interval since their last shower or bath. Height and weight were measured during routine physical exams [36]. Considering covariates is essential for the following reasons: (1) to control for confounding factors to minimize their impact on the outcome; (2) to improve the accuracy and reliability of the analysis; (3) to better reflect the true relationship between the independent and dependent variables; (4) to provide a comprehensive understanding of the phenomena under study; (5) to reduce bias from unconsidered covariates; and (6) to enhance the interpretability of results. The proper handling of covariates can improve the quality and credibility of research.

#### 2.5.6. Software Selection

For this meta-analysis, Review Manager 5.4 (RevMan) was chosen for data statistics and analysis. RevMan is a specialized software provided by the Cochrane Collaboration for systematic reviews and meta-analysis, serving as the standardized software for Cochrane systematic reviews [48]. RevMan offers several advantages: (1) data entry—supports the entering of relevant study information, such as study characteristics and outcome data; (2) statistical analysis: provides various meta-analysis methods, such as fixed-effects and random-effects models; (3) forest plot generation—visually presents results from individual studies and overall effects; (4) heterogeneity assessment—assists in evaluating variability between studies; (5) sensitivity analysis—tests the stability of results; and (6) publication bias detection—assesses whether publication bias exists. These features help in conducting systematic and standardized data analysis and synthesis. However, it is crucial to adhere to methodological principles and procedures to ensure the reliability and accuracy of the analysis results.

#### 2.5.7. Chart Creation

After installing the software, open RevMan and create a new worksheet by clicking “File—New” and selecting “Intervention review” in the “Type of review” page, and then click “Next”. Enter the study details in the subsequent dialog box. In the left sidebar, expand “Tables”, right-click on “Characteristics of studies”, and select “Add study” to input the names of the 7 included studies, typically formatted as “Author Year” (e.g., “Sun, Yang 2021”). Next, go back to the left sidebar, right-click on “Figures”, and choose the desired chart type. Import the required entries from the data organized from the 7 studies into the table to generate the desired flowchart, forest plot, or funnel plot for further analysis.

Understanding the functions of forest plots and funnel plots is essential for their effective use. Forest plots display the effect sizes and confidence intervals for each study, as well as the overall effect estimate and study heterogeneity. Funnel plots are used to assess publication bias, helping to detect whether there is any bias and to observe the symmetry of the research distribution.

## 3. Results

### 3.1. Covariate—Age

**Figure 4:**
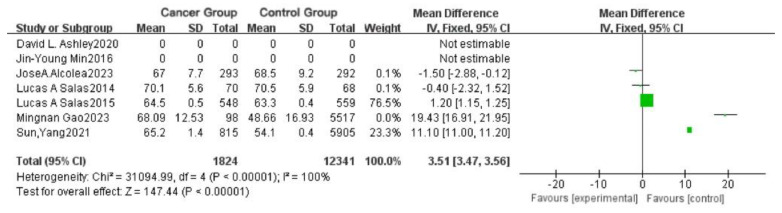
Forest plot of the difference in cancer incidence among different ages.

**Figure 5:**
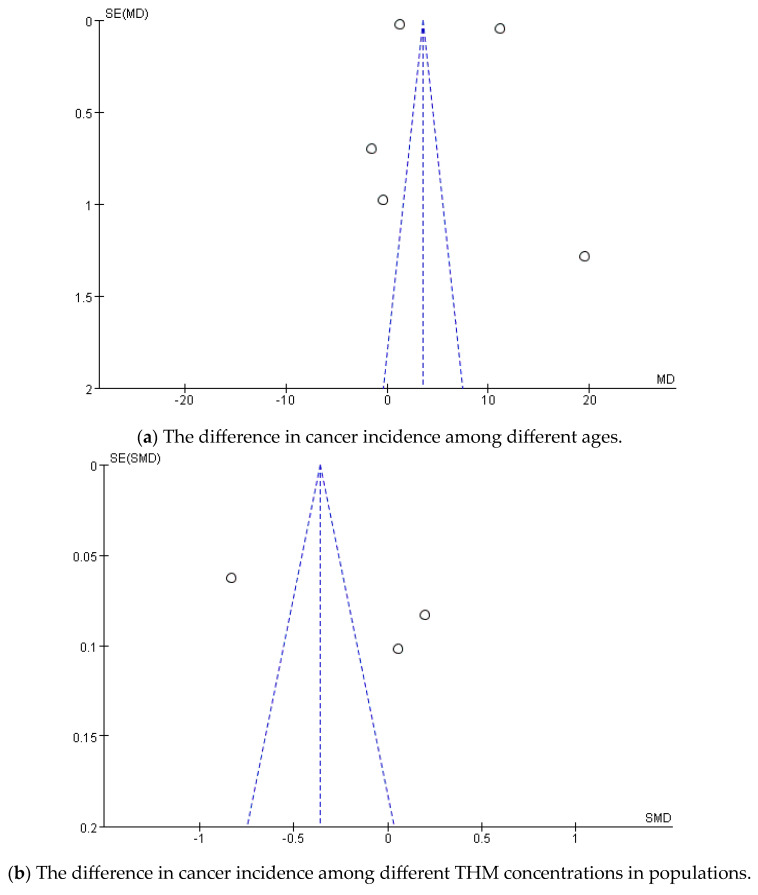
Publication bias: (**a**) the difference in cancer incidence among different ages; (**b**) the difference in cancer incidence among different THM concentrations in populations.

There were significant age range differences among the study populations included in this research; some studies required participants to be over 40 years old, while others only required participants to be over 20 years old. To minimize the potential influence of age on the accuracy of the experimental results, we first analyzed the participants’ ages, as shown in Figure 4 and Figure 5a.

As shown in Figure 4, after two studies with missing or invalid data are excluded, three groups have 95% confidence intervals (CIs) intersecting the null line, indicating that the mean differences in these groups were not statistically significant. The effect size (*p* < 0.00001, I^2^ = 100%) suggests high heterogeneity and substantial variation between studies, implying that age differences might indeed affect cancer incidence. The overall mean difference (MD) is 3.51, with a 95% CI [3.47, 3.56].

As depicted in Figure 5a, all data points fall outside the dashed lines, further indicating the high heterogeneity across these experiments. The distribution of studies on both sides of the vertical line, forming an inverted funnel shape, suggests no evidence of publication bias.

### 3.2. Statistical Analysis of THM Concentration

**Figure 6:**
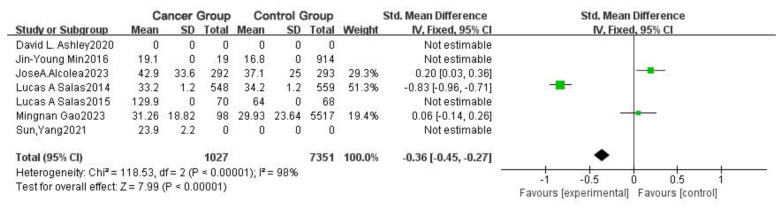
Forest plot of the difference in cancer incidence among different THM concentrations in populations.

In Figure 6, three groups provide valid data, with one showing no statistically significant difference in mean values. The overall standardized mean difference (SMD) is −0.36 [95% CI: −0.45, −0.27], *p* < 0.00001, with an I^2^ of 98%. This indicates that the mean concentration of THMs in the experimental group (patients with cancer) was lower than in the control group.

As shown in Figure 5b, the data points all fall outside the dashed lines, once again indicating high heterogeneity across the experiments. The distribution of studies on both sides of the vertical line in an inverted funnel shape further suggests no publication bias.

### 3.3. Analysis of Chloroform Concentration

**Figure 7:**
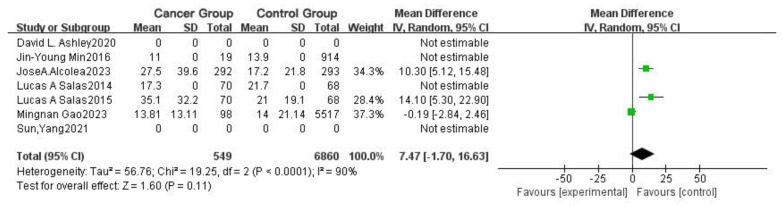
Forest plot of the difference in cancer incidence among different chloroform concentrations in blood in populations.

In Figure 7, the “diamond” intersects with the null line, indicating that the combined results of the control and experimental groups do not show statistical significance. Due to the limited number of studies included, it is not possible to conclusively determine the relationship between chloroform concentration in blood and cancer incidence based solely on these studies.

### 3.4. Sensitivity Analysis

A sensitivity analysis was performed of the above results. After each study was systematically excluded individually, no significant changes were observed in the conclusions, and there was no sharp reduction in heterogeneity.

## 4. Discussion

This meta-analysis aimed to assess whether there is a link between blood trihalomethane (THM) levels and cancer incidence. Through the integration of seven studies, some meaningful results were obtained.

Based on the forest and funnel plots generated using RevMan 5.4, our findings suggest a relationship between blood THM concentration and cancer incidence. Interestingly, the mean concentration of THMs in the blood of patients with cancer was lower than that of patients without cancer, which contrasts with some previous studies. This discrepancy may be attributed to two factors: first, the number of studies on this topic remains limited, and more research is needed to determine whether there is a positive or negative correlation; second, cancer development is likely influenced by other exposure factors. A further analysis of age showed that age differences indeed affect cancer incidence.

During the study, we employed strict criteria for data selection and extraction. We used explicit exclusion criteria to ensure that the included studies were relevant and reliable, thereby enhancing the reliability of our results. Each study was carefully assessed for methodological quality, including aspects such as study design, sample size, and data collection methods. In the quality control process, we paid special attention to data integrity and accuracy, ensuring consistency across studies. Most of the studies focused on recent (within three years) exposure windows rather than long-term exposure, as previous research has shown a higher correlation between recent exposure and biomarker effects compared to long-term exposure [49]. However, potential limitations remain, such as publication bias and variations in study quality. Furthermore, due to the temporal and geographical constraints of the included studies, the results may not fully represent the broader research field.

There is increasing evidence that THM exposure is associated with various cancers’ incidence and mortality rates [31,50,51]. After thoroughly reviewing the seven studies, in addition to extracting their research data, we also obtained valuable information. Besides chloroform, other THMs such as bromoform, BDCM, DBCM, TBM, and TCM may also influence cancer incidence depending on their concentration in the human body. In Mingnan Gao’s 2023 study [40], Poisson regression and subgroup analyses were used to evaluate the relationship between individual THM components and non-melanoma skin cancer (NMSC). After adjusting for covariates, Poisson regression showed that higher blood TBM levels were associated with an increased likelihood of NMSC (OR = 1.03; 95% CI: 1.01–1.05, *p* = 0.002), while the levels of TCM, DBCM, and BDCM were not significantly associated with NMSC (*p* > 0.05). Subgroup analysis and interaction tests revealed no significant differences in the association between TBM levels and NMSC based on age, gender, or race (all *p* > 0.05), suggesting a positive correlation between elevated TBM levels and NMSC [49]. Furthermore, exposure to high levels of THMs in drinking water has been linked to an increased mortality risk for colorectal, bladder, and brain cancers [36].

Currently, research on the carcinogenic mechanisms of THMs is relatively limited. The carcinogenicity of THMs involves several specific biological pathways. One critical pathway is the induction of oxidative stress, where THMs generate reactive oxygen species (ROS) that cause DNA damage, protein oxidation, and lipid peroxidation [52]. This oxidative stress can activate the nuclear factor kappa-light-chain-enhancer of activated B cells (NF-κB) pathway, which plays a key role in inflammation and cancer progression [53]. Another significant pathway is the formation of DNA adducts, particularly in bladder epithelial cells, where brominated THMs are metabolized into reactive intermediates that bind to DNA, leading to mutagenesis and carcinogenesis [37,54,55]. Additionally, THMs may contribute to carcinogenesis through endocrine disruption and the disruption of normal cell cycle control [56,57,58]. These pathways highlight the multifaceted nature of THM-induced carcinogenesis, underscoring the need for further research to fully elucidate these mechanisms. These specific mechanisms underscore the complex biological impacts of THMs on cancer development across different tissues and warrant further investigation in the future. Moreover, it remains unclear whether there are differences in carcinogenic mechanisms among different types of THMs, variations in their accumulation and target sites within the body, and differences in the types of cancers they may induce, necessitating the acquisition of these data in future studies.

## 5. Limitations and Future Directions

### 5.1. Limitations

While this meta-analysis provides valuable insights, it has several limitations: (1) The number of studies available for inclusion was small, which may have introduced publication bias. Unpublished studies could potentially influence the results. Moreover, specific cancer types were not separately analyzed. (2) The quality of the included studies varied, which could affect the reliability of the results. (3) The potential heterogeneity could not be fully explained, as there were insufficient data on covariates such as gender, race, and lifestyle habits, which limited further analysis. (4) There were inconsistencies in the data across studies, and the available data were significantly reduced after screening, leading to a decrease in the precision of the experimental results. Although a correlation between THMs and cancer was observed, the mechanisms of THM-induced carcinogenesis in humans remain unclear. There is still insufficient evidence to determine the importance of specific THMs in relation to cancer, and it is not yet clear whether the observed associations are causal. Future research is needed to clarify these issues.

### 5.2. Future Directions

Despite these limitations, this meta-analysis offers important value and potential for future research. In terms of research methodology, future studies should aim to improve the quality of included studies, address heterogeneity more rigorously, and strengthen the identification and control of potential biases. Combining meta-analysis with other research methods may provide a more comprehensive understanding. More targeted meta-analyses should also be conducted to provide stronger evidence for the development of this field. In terms of research content, more prospective studies are needed to explore the relationship between the monitoring of blood THM levels and the early prevention of various cancers. Additionally, future research should focus on analyzing specific cancer types separately to uncover critical nuances in the relationship between THM exposure and different cancer risks, which could be overlooked due to the small number of available studies. Conducting similar studies using animal models could provide a more controlled environment, yielding more reliable results and additional insights. Incorporating data from such animal studies alongside data from human studies would offer a more comprehensive understanding of the effects of THMs on cancer development, ultimately strengthening the findings and enhancing the depth of knowledge in this area.

## 6. Conclusions

This meta-analysis confirms an association between blood THM concentrations and cancer incidence, with age-related variations observed. The analysis of age-related covariates revealed significant heterogeneity, suggesting that age differences among study populations could influence cancer risk, with an MD of 3.51 in cancer incidence across different age groups, indicating a potential age-related effect. Statistical analysis showed that patients with cancer had lower mean THM levels than controls, with an SMD of −0.36, despite high heterogeneity, suggesting a complex relationship between THM exposure and cancer risk. The analysis specific to chloroform did not yield statistically significant results, highlighting the need for further research with larger sample sizes to clarify this association. Sensitivity analyses confirmed the robustness of the findings, as no substantial changes were observed upon the exclusion of individual studies. This meta-analysis deepens the understanding of the relationship between THM exposure and cancer while emphasizing the need for additional research to address heterogeneity and to further explore the mechanisms underlying THM-induced carcinogenesis, providing a foundation for future investigations into the public health implications of exposure to THMs, as a significant class of disinfection by-products.

## Figures and Tables

**Table 1:** Summary table of included articles.

First Author	Year	Region	Study Population		Average Age of Case Group	Conclusion	Cancer Type
			Total Number	Number of Cases			
Cohort Studies							
Sun, Yang [36]	2021	USA	6720	815	65.2	There was a positive dose–response relationship between blood levels of DBCM and TBM and the risk of cancer mortality.	All cancers
Lucas A. Salas [37]	2015	Spain	138	70	70.1	Long-term exposure to THMs affects DNA methylation of tumor-related genes.	Colorectal cancer
Cross-sectional Studies							
Jin-Young Min [38]	2016	USA	933	19	Unknown	THMs, especially brominated THMs, may be associated with increased cancer mortality.	All cancers
David L. Ashley [39]	2020	USA	12,171	942	Unknown	Bladder cancer is associated with long-term exposure to THMs.	Bladder cancer
Mingnan Gao [40]	2023	China	5715	98	68.09	Elevated levels of TBM in the blood were positively correlated with NMSC risk in adults aged 65 and above.	Non-melanoma skin cancer (NMSC)
Case–Control studies							
Lucas A. Salas [41]	2014	Spain	1107	559	63.3	There was a positive correlation between LINE-1 %5mC levels and THM concentrations in the control group.	Bladder cancer
Jose A. Alcaide [42]	2023	Spain	585	292	68.5	THMs and chloroform were significantly higher in cancer cases than in controls.	Colorectal cancer

## Data Availability

This is a meta-analysis paper, and all of the data were collected from published papers.
